# Hip Fracture Mortality: Is It Affected by Anesthesia Techniques?

**DOI:** 10.1155/2012/708754

**Published:** 2012-01-12

**Authors:** Saffet Karaca, Egemen Ayhan, Hayrettin Kesmezacar, Omer Uysal

**Affiliations:** ^1^Department of Anesthesiology and Reanimation, Cerrahpasa Medical Faculty, Istanbul University, 34098 Istanbul, Turkey; ^2^Department of Orthopaedics and Traumatology, Sariyer Ismail Akgun Public Hospital, 34473 Istanbul, Turkey; ^3^Department of Orthopaedics and Traumatology, Istanbul Bilim University Medical Faculty, 34349 Istanbul, Turkey; ^4^Department of Biostatistics and Medical Informatics, Bezmialem Vakif University Medical Faculty, 34093 Istanbul, Turkey

## Abstract

We hypothesized that combined peripheral nerve block (CPNB) technique might reduce mortality in hip fracture patients with the advantage of preserved cardiovascular stability. We retrospectively analyzed 257 hip fracture patients for mortality rates and affecting factors according to general anesthesia (GA), neuraxial block (NB), and CPNB techniques. Patients' gender, age at admission, trauma date, ASA status, delay in surgery, followup period, and Barthel Activities of Daily Living Index were determined. There were no differences between three anesthesia groups regarding to sex, followup, delay in surgery, and Barthel score. NB patients was significantly younger and CPNB patients' ASA status were significantly worse than other groups. Mortality was lower for regional group (NB + CPNB) than GA group. Mortality was increased with age, delay in surgery, and ASA and decreased with CPNB choice; however, it was not correlated with NB choice. Since the patients' age and ASA status cannot be changed, they must be operated immediately. We recommend CPNB technique in high-risk patients to operate them earlier.

## 1. Introduction

“Hip fracture” refers to a fracture of the femur in the area of bone immediately distal to the articular cartilage of the hip, to a level of about five centimeters below the lower border of the lesser trochanter [1]. Hip fracture prevalence is rising with the continued ageing of the population [2]. Studies have demonstrated the increased risk of mortality after hip fracture especially during the first year, and excess mortality risk may persist for several years after fracture [3–5]. 23.8% of patients die in the first year after hip fracture and one in three patients require a higher level of long-term care [3].

For hip fracture operations, besides the general anesthesia (GA) and neuraxial block (NB) techniques, recently, the combined lumbar plexus and sciatic nerve block (CLSB) technique is recommended, especially for high-risk patients [6–10]. When compared with GA and NB, minimal hemodynamic disturbance and so less affected cardiovascular stability are the advantages of CLSB [6–11]. NB is argued to reduce mortality when compared with GA [1, 12, 13]; however, survival studies in hip fracture patients have not analyzed the effects of CLSB on mortality.

In our recently published research about mortality after hip fracture [14], there was an uncertain relationship between mortality and anesthesia type. In order to face the relationship out, we purposed to determine mortality of patients after hip fracture according to anesthesia type. Considering the preserved cardiovascular stability with CLSB technique, we hypothesized that CLSB choice might reduce mortality.

## 2. Materials and Methods

This study is approved by Istanbul University, Cerrahpasa Medical Faculty Research Ethics Committee. The records of all patients who underwent hip fracture surgery at our institution between January 1, 2000 and December 31, 2007 were reviewed. Previously ambulatory 65 years and older patients are included. All of the living patients were followed up for at least one year. Cancer patients and patients with insufficient preoperative data were excluded. Two hundred fifty-seven patients were included in the study.

The patients were divided into three groups according to anesthesia type as general anesthesia group (GA), neuraxial block group (NB), and combined peripheral nerve block group (CPNB). CPNB term was preferred to define addition of lateral femoral cutaneous nerve block to CLSB.

Patients' anesthesia types were evaluated by anesthesiology charts and data. Gender, age at admission, trauma date, and days passed until surgery were obtained from patients' computerized data, hospital charts, and folders. All of the patients were prescribed low-molecular-weight heparin for anticoagulation from admission to hospital to postoperative 20 days. Patients were phoned for followup and questioned for activity status. If a patient was not available for followup, a family member was interviewed; if the patients were dead, date of death; if they survived, daily living activity questioned. Daily living activity was scored by using Barthel Activities of Daily Living Index.

The preoperative status of the patients was classified according to the American Society of Anesthesiologists' (ASA) physical scale status to predict operative risk.

### 2.1. Types of Anesthesia

1. GA: endotracheal anesthesia achieved by intravenous drugs (propofol and fentanyl), neuromuscular blockers (atracurium), and inhalation agents (sevoflurane) to render the patient unconscious.
2. NB: by injection of local anesthetic (bupivacaine) into the epidural or subarachnoid spaces.
  - Epidural anesthesia: an epidural catheter was placed, and 10 mL bupivacaine 0.5% isobaric were injected by this catheter. If necessary, 2 mL bupivacaine of incremental doses were injected during the perioperative course.
  - Spinal anesthesia: bupivacaine 0.5% isobaric 7.5–15 mg was used for local anesthetic agent.
3. CPNB: posterior lumbar plexus block, posterior sciatic block, and lateral femoral cutaneous nerve block [15–17].
  - Lumbar plexus block: 15 mL Prilocain 2% + 15 mL bupivacaine 0.5%.
  - Sciatic block: 10 mL Prilocain 2% + 10 mL bupivacaine 2%.
  - Lateral femoral cutaneous nerve block: 10 mL Lidocaine 2%.

### 2.2. Statistical Analysis

The unadjusted *χ* ^2^ test was used for analyzing differences between proportions. The one-way ANOVA test was used for analyzing differences between means of three groups. The ASA status among three groups was compared with Kruskal-Wallis test. To compare the groups' median score of ASA status with each other, Mann-Whitney test was used.

The cumulative survival rates were obtained as Kaplan-Meier estimates, and the log rank test was used to find *P* value. To determine the association between potential predictors and mortality, Cox proportional hazards regression was used. *P* < 0.05 was defined to be significant in all tests.

## 3. Results

Two hundred fifty-seven patients met the inclusion criteria and were included in the study. There were three groups of patients according to anesthesia techniques: 115 patients with GA, 50 patients with NB, and 92 patients with CPNB. The baseline characteristics of the study population according to anesthesia techniques are summarized in Table 1. There were no significant differences between three groups regarding to sex, mean followup, delay in surgery, and Barthel score. The patients mean age was 80.6 ± 8.3 for GA, 77.1 ± 7.8 for NB, and 81.0 ± 7.4 for CPNB (*P* = 0.013). NB group was significantly younger than the other two groups.

The ASA status among three groups was significantly different (*P* < 0.001) with Kruskal-Wallis test. To compare the groups' ASA status with each other, Mann-Whitney test was used. There were no significant differences in the ASA status between GA-NB (*P* = 0.2599). However, the ASA status were significantly different between CPNB and GA (*P* < 0.001), and between CPNB and NB (*P* < 0.014). CPNB patients' health status was worse than the other groups. The ASA status of patients according to groups is shown thoroughly in Table 2.

### 3.1. Mortality

**Figure 1:**
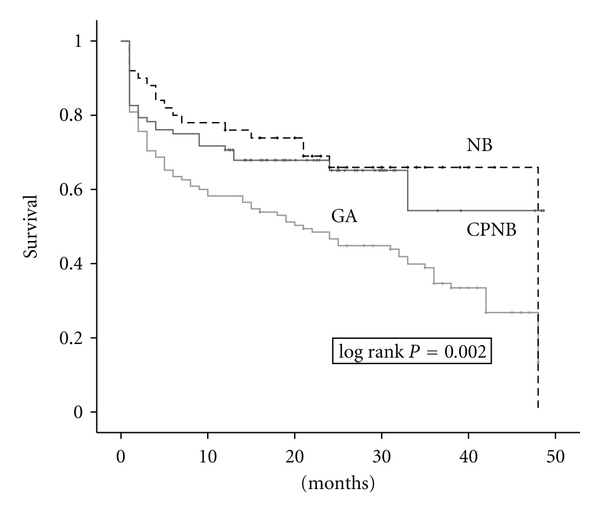
The graph shows a Kaplan-Meier survival curve for general anesthesia (GA), neuraxial block (NB), and combined peripheral nerve block (CPNB) patients.

The one-month mortality rates of GA patients, NB patients, and CPNB patients were 19.1%, 8%, and 17.4%, respectively (*P* = 0.195). The one-year mortality rates of GA patients, NB patients, and CPNB patients were 41.7%, 22%, and 28.3%, respectively (*P* = 0.022). One-year mortality rate was significantly lower for regional group (NB + CPNB) than GA group. The overall mortality rates of GA patients, NB patients, and CPNB patients were 69.6%, 36%, and 33.7%, respectively (*P* < 0.001). Overall mortality rate was significantly lower for regional group (NB + CPNB) than GA group. Estimated mean survival time for GA patients, NB patients, and CPNB patients was 23.4 ± 1.8 months, 34.6 ± 2.8 months, and 31.8 ± 2.6 months, respectively. Estimated mean survival time was significantly higher for regional group (NB + CPNB) than GA group (*P* = 0.002). Mortality rates are summarized in Table 1, and survival curves are presented in Figure 1.

To determine the association between potential predictors (age, sex, ASA status, delay in surgery), anesthesia type and mortality, Cox regression analysis was used. In the first Cox regression analysis GA was categorized as reference group, and NB and CPNB anesthesia types, were taken as variables in regards to the reference, GA group. Age (*P* = 0.008), delay in surgery (*P* = 0.021), and ASA (*P* = 0.033) were found as significant predictors of mortality. Both NB and CPNB choices were found to decrease mortality in this multivariate analysis. Since the anesthesia types were nominal variables in three different categories, we performed two more Cox regression analyses in order to find out the distinction between NB and CPNB choices. In the second Cox regression analysis, GA and NB groups were collectively assigned as reference in regards to CPNB variable. CPNB was shown to decrease mortality significantly (*P* = 0.029, odds ratio = 0.627). However, in the third Cox regression analysis, NB was not correlated with decreased mortality (*P* = 0.068), when GA and CPNB groups were collectively assigned as reference in regards to NB variable. Cox regression analyses are shown in Table 3 in details.

### 3.2. Functional Outcome

For CPNB patients (*n* = 61), the mean of the Barthel score was 14.9, for the NB patients (*n* = 33), it was 14.3, and for GA patients (*n* = 41), it was 14.4 (*P* = 0.887). There was no significant difference between three groups.

## 4. Discussion

We retrospectively analyzed 257 hip fracture patients to determine mortality rates and factors affecting patient mortality, according to three anesthesia techniques.

ASA physical scale status is commonly used to classify the preoperative status of the hip fracture patients [18–20]. Hamlet et al. [18] reported that 3-year mortality was significantly less for ASA I and II patients (23%) than for ASA III, IV, and V patients (39%). Michel et al. [19] reported that in 114 patients treated for hip fracture, high ASA status (3 or 4) conferred a nine times increased risk for mortality at one year. However, in the review for anesthetic risk factors, Haljamäe [21] stated that because ASA classification considers only physical status factors, other risk-predictive factors such as age and sex of the patient and the type, site, and duration of surgery should also be included for individual cases. Our patients' hip fractures were either femoral neck or intertrochanteric femur fracture. Because of different surgery modalities for these fractures, we could not take into consideration the perioperative blood loss and duration of surgery, that are the major limitations of our study. Also, in our recent research about predictors of mortality after hip fracture [14], we did not find any relationship between comorbidities (systemic diseases) and mortality. So, rather than the quantity (count), we preferred the significance of the diseases, which is reflected better with ASA. But, besides ASA, we included the age, sex, and delay in surgery as risk factors for mortality in multivariate analysis. We found that ASA, age, and delay in surgery were significant predictors of mortality.

When the three groups of patients were compared, there were no significant differences for sex, delay in surgery, mean followup, and Barthel score. Similar to other studies [12, 22, 23], delay in surgery is associated with increased mortality in this study, but has no emphasis for comparison of these three groups' mortality. However, the mean age of the NB patients was significantly younger than GA and CPNB patients, which would decrease the mortality of NB patients [2, 4, 24–26]. Also, the ASA status of CPNB patients was significantly worse than GA and NB patients, that would increase the mortality of CPNB patients according to other studies [18–20].

The one-month mortality rate was not significantly different for the three (GA, NB, and CPNB) groups. However, both one-year and overall mortality rates were decreased for the regional group (NB + CPNB). Also estimated survival time was higher for regional group. In several studies, the reduction in morbidity and mortality had been shown with regional anesthesia [12, 13]. Although there was no significant difference, the one-month mortality rates were 19.1%, 8%, and 17.4% for GA, NB, and CPNB patients, respectively. We believe that the younger mean age and better ASA status of NB patients than CPNB patients caused this one-month difference. However, by the time, if the highrisk patients succeeded in surviving for one month, the survival rate of CPNB patients became almost equal to the NB patients, even though they were older than the NB patients (Figure 1). Confirming this, CPNB choice was an independent variable of decreased mortality; however, NB choice was not in multivariate Cox regression analyses (Table 3).

Naja et al. [10] treated 60 patients for hip fracture, 30 patients with general anesthesia, and 30 patients with combined sciatic-paravertebral nerve block. They reported that both the incidence of intraoperative hypotension and the postoperative need for intensive care unit admission was significantly reduced in patients treated with combined sciatic-paravertebral nerve block compared to patients receiving general anesthesia. Similarly, in their prospective randomized study, de Visme et al. [6] treated 29 patients for hip fracture, 15 patients received combined lumbar and sacral plexus block, and 14 patients received spinal anesthesia. They found that hypotension was to be longer lasting after spinal anesthesia and of a larger magnitude in patients over 85 years of age. CLSB, as a rising trend, is correlated with minimal hemodynamic disturbance and so less affected cardiovascular stability [6–11]. These advantages of CPNB promote us to operate high-risk (ASA III AND IV) hip fracture patients earlier without seeking medical treatment modalities for their systemic diseases.

In conclusion, to decrease the mortality rate after hip fracture, since age and ASA status are patient-dependent factors that cannot be changed, the patients must be operated as soon as possible. Because CPNB is an encouraging technique to operate patients earlier, we recommend CPNB technique in hip fracture patients, especially for patients with poor general health status. Considering the retrospective nature of the study and the effects of personal characteristics, it is hard for us to claim that “*CPNB technique decreases mortality.”* Nevertheless, our hypothesis and results at least may form the basis and show the need for future randomized prospective studies.

## Figures and Tables

**Table 1:** Characteristics of the study population according to anesthesia type.

Characteristics	GA (*n* = 115)	NB (*n* = 50)	CPNB (*n* = 92)	*P* value
Sex (M/F)	40/75	17/33	23/69	0.284
Age	80.6 ± 8.3	77.1 ± 7.8	81 ± 7.4	0.013*
Delay in surgery (Days)	10 ± 9.12	12.1 ± 11.2	8.7 ± 6.2	0.056
Followup (months)	21 ± 17.4	21.9 ± 13.3	17 ± 12.2	0.09
Barthel score	14.4 ± 6.4	14.3 ± 6.9	14.9 ± 5.2	0.887
One-month mortality	22 (19.1%)	4 (8.0%)	16 (17.4%)	0.195
One-year mortality	48 (41.7%)	11 (22%)	26 (28.3%)	0.022**
Overall mortality	80 (69.6%)	18 (36%)	31 (33.7%)	0.001^†^
Estimated survival (months)	23.4 ± 1.8	34.6 ± 2.8	31.8 ± 2.6	0.002^‡^

*P < 0.05, NB group is significantly younger than the other groups.

**P < 0.05, one-year mortality rate of regional group (NB + CPNB) is significantly reduced compared to GA group.

^†^ *P* < 0.01, overall mortality rate of regional group (NB + CPNB) is significantly reduced compared to GA group.

^‡^ *P* < 0.01, estimated mean survival time is significantly higher for regional group (NB + CPNB) than GA group.

GA: general anesthesia, NB: neuraxial block, and CPNB: combined peripheral nerve block.

**Table 2:** ASA status of patients.

	GA (*n* = 115)	NB (*n* = 50)	CPNB (*n* = 92)
ASA I	2 (1.7%)	0 (0%)	0 (0%)
ASA II	32 (27.8%)	10 (20.0%)	5 (5.4%)
ASA III	72 (62.6%)	36 (72.0%)	74 (80.4%)
ASA IV	9 (7.8%)	4 (8%)	13 (14.1%)
Mean ASA	2,7652	2,8800	3,0870

ASA comparison		*P* < 0.001	
GA-NB-CPNB*			

ASA comparison		*P* = 0.259	
GA-NB			

ASA comparison		*P* = 0.014	
NB-CPNB*			

ASA comparison		*P* < 0.001	
GA-CPNB*			

*P < 0.05: CPNB patients' ASA score is significantly worse than GA and NB patients.

**Table 3:** Summary of Cox regression analyses.

Variable	Significance	Odds ratio
Age	0.008*	1.030
Sex	0.287	0.820
ASA status	0.033*	1.432
Delay in surgery	0.021*	1.020
Anesthesia type	0.003*	
GA versus CPNB (Cox 1)	0.005*	0.537
GA versus NB (Cox 1)	0.012*	0.508
GA + NB versus CPNB (Cox 2)	0.029*	0.627**
GA + CPNB versus NB (Cox 3)	0.068	0.619

*P < 0.05.

**Odds ratio <1 is associated with decreased hazard of the event (in this case “CPNB choice is associated with decreased mortality”).
